# Clinical and microbiological characteristics of pediatric patients hospitalized for pneumococcal pneumonia before and after the introduction of pneumococcal conjugate vaccines

**DOI:** 10.17843/rpmesp.2025.421.13847

**Published:** 2025-03-12

**Authors:** Carolina A. Gomez, Brayan E. Gonzales, Roger A. Hernández, Francisco Campos, Eduardo Chaparro, Olguita Del Águila, María E. Castillo, Andrés Saenz, Isabel Reyes, Theresa J. Ochoa

**Affiliations:** 1 Faculty of Human Medicine “Alberto Hurtado”, Universidad Peruana Cayetano Heredia, Lima, Peru. Faculty of Human Medicine “Alberto Hurtado” Universidad Peruana Cayetano Heredia Lima Peru; 2 Alexander von Humboldt Institute of Tropical Medicine, Universidad Peruana Cayetano Heredia, Lima, Peru. Alexander von Humboldt Institute of Tropical Medicine Universidad Peruana Cayetano Heredia Lima Peru; 3 Department of Pediatrics, Cayetano Heredia National Hospital, Lima, Peru. Department of Pediatrics Cayetano Heredia National Hospital Lima Peru; 4 San Bartolomé National Teaching Hospital for Mothers and Children. Lima, Peru. San Bartolomé National Teaching Hospital for Mothers and Children Lima Peru; 5 Clinical Specialty Pediatrics Service, Edgardo Rebagliati Martins National Hospital, Lima, Peru. Clinical Specialty Pediatrics Service Edgardo Rebagliati Martins National Hospital Lima Peru; 6 Epidemiology Office, National Institute of Child Health, Lima, Peru. Epidemiology Office National Institute of Child Health Lima Peru; 7 Department of Pediatrics, Daniel Alcides Carrión National Hospital, Lima, Peru. Department of Pediatrics Daniel Alcides Carrión National Hospital Lima Peru; 8 Hospitalization Service, Pediatric Emergency Hospital, Lima, Peru. Hospitalization Service Pediatric Emergency Hospital Lima Peru

**Keywords:** Pneumococcal Pneumonia, Pneumococcal Vaccines, Empyema, Children, Lima

## Abstract

The clinical and microbiological characteristics of pneumococcal pneumonia in children hospitalized in Lima were analyzed, before and after the introduction of pneumococcal conjugate vaccines (PCV). We reviewed cases that occurred between 2006-2019, in the pre-PCV7 (2006-2008), post-PCV7 (2009-2011) and post-PCV13 (2016-2019) periods. Of 128 patients with positive cultures (92 blood and 36 pleural fluid), most were infants in the pre-PCV7 (77.1%) and post-PCV7 (43.3%) studies, while in the post-PCV13 study they were mostly preschoolers (62%). We found an increase in complicated pneumonia cases from 14.6% to 72% and an increase in serotype 19A (6.3% to 68%). The most frequent sequence type was ST320 (31 isolates). Resistance to penicillin and ceftriaxone was low (6% and 2%, respectively), although higher in empyema (21.4% and 14.3%, respectively). Resistance to azithromycin increased from 27% to 92%. Penicillin remains the antibiotic of choice for the treatment of uncomplicated pneumococcal pneumonia in Lima.

## INTRODUCTION

Pneumonia is the leading infectious cause of death in children under five years of age, accounting for 14% of deaths in this age group. *Streptococcus pneumoniae*, or pneumococcus, is the leading cause of bacterial pneumonia in children ^1^. In 2015, 9.18 million cases of pneumococcal infection were reported globally in the general population, with 318,000 fatal cases. Approximately 97% of pneumococcal infections are pneumonia, while 81% of deaths caused by pneumococcus are due to pneumonia ^2^.

The use of pneumococcal conjugate vaccines (PCV) as a public health measure has changed the dynamics of this disease. Worldwide, from 2000 to 2015, cases of pneumonia in children decreased in 37% after the introduction of vaccines ^2^. In Peru, the first vaccine to be used was PCV7 in 2009, followed by PCV10 in 2011 and finally PCV13 in 2015 ^3^. PCV15 and PCV20 are not yet available in Peru. The distribution of pneumococcal serotypes varies according to region and the PCVs introduced ^4^.

Studies conducted in Lima over the past 14 years have allowed for comparisons of the resistance and distribution of invasive pneumococcal disease (IPD) serotypes. However, no analysis focused specifically on cases of pneumonia. This study aimed to describe and compare the clinical, demographic, and microbiological characteristics of pediatric patients with pneumococcal pneumonia before and after the introduction of PCV in Lima, Peru.

**KEY MESSAGES**

Motivation for the study. Although studies on invasive pneumococcal disease have been conducted in Lima, the characteristics of pneumococcal pneumonia before and after the introduction of pneumococcal conjugate vaccines are unknown.

Main findings. We found an increase in complicated pneumococcal pneumonia cases, particularly empyema and an increase in serotype 19A. Resistance to penicillin and ceftriaxone was found to be low; however, in cases of empyema, antibiotic resistance was higher.

Public health implications. Penicillin or amoxicillin should continue to be prescribed for the empirical management of pneumococcal pneumonia; however, ceftriaxone is recommended for cases with empyema. Continued local monitoring of pneumococcal resistance and serotypes is recommended.

## THE STUDY

### Study design

Descriptive study of secondary analysis of databases obtained from the medical records of three multicenter passive surveillance studies of IPD conducted by the Peruvian Pneumococcal Research Group (GPIN) in the periods 2006-2008 [pre-PCV7 (ENI-1)] ^5^, 2009-2011 [post-PCV7(ENI-2)] ^6^ and 2016-2019 [post-PCV13(ENI-3)] ^7^. The studies were conducted in 11 public hospitals and 7 private clinics and laboratories in Metropolitan Lima.

These studies collected clinical information, antibiotic resistance, and genomic data. The E-test method was used to determine the minimum inhibitory concentration (MIC) and Kirby-Bauer for 12 antibiotics, using the cut-off points established by CLSI-2019 ^8^ (supplementary table 1). Serotypes and sequence types (STs) were determined by whole genome sequencing (WGS).

### Population

The population consisted of patients under the age of 18 hospitalized in Lima and diagnosed with pneumococcal pneumonia.

### Case definition

Pneumococcal pneumonia was defined as the isolation of *S. pneumoniae* in blood culture or pleural fluid (PF) culture in patients with an infectious process with fever, respiratory distress, and/or evidence of pulmonary infiltrates on chest X-ray. Complicated pneumonia was defined in the context of pneumococcal pneumonia with the addition of local complications (pleural effusion, empyema, necrotizing pneumonia, respiratory failure requiring mechanical ventilation) or systemic complications (sepsis or death) ^6^.

### Variables

The variables are described in supplementary table 3.

### Statistical analysis

Categorical variables are reported as absolute and relative frequencies, and continuous variables as medians and interquartile ranges (IQR). The Chi-square test of independence or Fisher’s exact test was used to determine differences between IPD studies, as appropriate. The Kruskall-Wallis test was used for continuous variables. All analyses were performed in STATA SE version 18.

### Ethical considerations

The IPD studies were approved by the Research Ethics Committee of the Cayetano Heredia Peruvian University (UPCH) and by each participating hospital. This study was approved by the UPCH Research Ethics Committee (SIDISI 211822).

## RESULTS

We identified 128 cases of pneumococcal pneumonia in pediatric patients, 48 in the pre-PCV7 period, 30 in the post-PCV7 period, and 50 in the post-PCV13 period. During the first two periods more cases of pneumococcal pneumonia in infants (77.1% and 43.3%, respectively) were reported, on the other hand, during the last period more cases in preschoolers (62%) were found. Of the total strains, 71.9% were isolated from blood cultures and 28.1% from LP; isolation in LP increased from 14.6% to 48%. In the post-PCV7 period, only 16.7% were vaccinated, while in the post-PCV13 study, 70% had received PCV10 or PCV13 (Table 1).

Although no data on complications were collected during the pre-PCV7 period, seven patients were identified with pleural effusion due to pneumococcal isolation from LP. We did find an increase in complicated pneumonia from 14.6% to 72%. The most frequent complication was empyema (35%), followed by sepsis and respiratory failure requiring mechanical ventilation. Among the 36 cases of post-PCV13 pneumococcal pneumonia, 25% were in patients with complete vaccination and 6 cases were due to serotype 19A (Table 1).

The frequency of serotype 19A increased from 6.3%, 13.3%, and 68.0%. Serotype 24F was not present in the first two periods, but during the post-PCV13 period it accounted for 22% of isolates. In addition, we found a decrease in serotype 14 (31.3%, 26.7%, and 0%). Among all isolates, the most frequent sequence types were ST320 (26.7%) and ST156 (12.9%) (Table 2).

**Table 1:** Demographic, clinical, and laboratory characteristics of pediatric patients with pneumococcal pneumonia a.

Characteristics		IPD-1 n (%)	IPD -2 n (%)	IPD -3 n (%)	Total n (%)
Sex, male (n=128)		28 (62.2)	16 (53.3)	22 (44.0)	66 (52.8)
Age, years ^b^, median (IQR)		1 (1-2)	2 (1-7)	2 (1-3)	1.5 (1-3)
Age group (n=128)					
	Infants (<2 years)	37 (77.1)	13 (43.3)	17 (34.0)	67 (52.3)
	Preschoolers (2-6 years)	10 (20.8)	7 (23.3)	31 (62.0)	48 (37.5)
	Schoolchildren (>6 years)	1 (2.1)	10 (33.3)	2 (4.0)	13 (10.2)
Comorbidity (n=40)		ND	16 (53.3)	24 (48.0)	40 (50.0)
	Malnutrition	ND	8 (26.7)	8 (16.0)	16 (20.0)
	Asthma	ND	4 (13.3)	3 (6.0)	7 (8.8)
	Anemia	ND	1 (6.3)	5 (10.0)	6 (7.5)
	Other ^c^	ND	3 (3.3)	6 (12.0)	11 (13.8)
PCV vaccination status (n=120)					
	Complete	0	2 (6.7)	25 (50.0)	27 (21.1)
	Incomplete	0	3 (10.0)	14 (28.0)	17 (13.3)
	None	48 (100.0)	22 (73.3)	6 (12.0)	76 (59.4)
Type of vaccine (n=118)					
	PCV13	0	0	21 (42.0)	21 (16.4)
	PCV10	0	0	14 (28.0)	14 (10.9)
	PCV7	0	5 (16.7)	2 (4.0)	7 (5.5)
	None	48 (100.0)	22 (73.3)	6 (12.0)	76 (59.4)
Culture site (n=128)					
	Blood	41 (85.4)	25 (83.3)	26 (52.0)	92 (71.9)
	Pleural fluid	7 (14.6)	5 (16.7)	24 (48.0)	36 (28.1)
Radiological pattern (n=67)					
	Alveolar	ND	15 (57.7)	34 (82.9)	49 (73.1)
	Alveolar-interstitial	ND	10 (38.5)	4 (9.8)	14 (20.9)
	Interstitial	ND	1 (3.9)	3 (7.3)	4 (6.0)
Laboratory tests					
	Leukocytes, median (IQR)	ND	7200 (3060-17 200)	6000 (3020-14 500)	6000 (3060-15 000)
	Segmented %, median (IQR)	ND	76 (64.75-87.25)	80 (75-85)	80 (70-90)
Complications ^d,e^ (n=50)		7 (14,6)	7 (23.3)	36 (72.0)	50 (39.1)
	Empyema	ND	3 (10.0)	25 (50.0)	28 (35.0)
	Sepsis	ND	2 (6.7)	17 (34.0)	19 (23.8)
	Respiratory failure	ND	2 (6.7)	17 (34.0)	19 (23.8)
	Necrotizing pneumonia	ND	1 (3.3)	13 (26.0)	14 (17.5)
	Pleural effusion	ND	1 (3.3)	6 (12.0)	7 (8.8)
Lethality (n=10)		7 (14.6)	0 (0.0)	3 (6.0)	10 (7.8)^f^

a Some variables total less than 128 due to missing values, which is why some percentages do not add up to 100%.

b p<0.001, for the comparison of IPD-1, IPD -2, and IPD -3 using the Kruskall-Wallis test.

c Other less frequent comorbidities: bronchial obstructive syndrome, cancer, congenital heart disease, chronic respiratory disease, prematurity, obesity, HIV. The percentages in the total column are calculated based on the total number of IPD-2 and IPD-3 patients.

d A patient may have more than one complication, so the percentages do not add up to exactly 100%. The most frequent presentations were six patients with empyema, necrotizing pneumonia, respiratory failure requiring mechanical ventilation, and four patients with empyema and respiratory failure requiring mechanical ventilation. For the distribution of complications in the total column, only IPD -2 and IPD -3 samples with data on the type of complication were considered (denominator n=80).

e p<0.001, for the comparison of IPD -1, IPD -2, and IPD -3 using the chi-square test of independence.

ND: Data not collected during the study, IQR: interquartile range, PCV: pneumococcal conjugate vaccines

f Percentage calculated from the total=128

**Table 2:** Distribution of serotypes and sequence types (ST) present in conjugate vaccines in patients with pneumococcal pneumonia (N = 128) ^a^.

		IPD-1 n=48 n (%)	IPD-2 n=30 n (%)	IPD-3 n=50 n (%)	Total n=128 n (%)	ST n=116^b^ ST (n)
Serotypes						
	19A^c^	4 (8.3)	4 (13.3)	34 (68.0)	42 (32.3)	320 (31) 276 (3) 1131 (2) 1451 (2) 5460 (1) 6048 (1)
	14	15 (31.3)	8 (26.7)	0 (0.0)	23 (18.0)	156 (15) 15 (2) 6144 (1) 7432 (1) 9054 (1)
	6B	12 (25.0)	4 (13.3)	0 (0.0)	16 (12.5)	90 (4) 5625 (3) 1121 (2) 135 (2) 1662 (1) 5449 (1) 5619 (1)
	24F	0 (0.0)	0 (0.0)	11 (22.0)	11 (8.6)	230 (9)
	5	4 (8.3)	2 (6.7)	0 (0.0)	6 (4.7)	289 (6)
	23F	2 (4.2)	3 (10.0)	0 (0.0)	5 (4.0)	242 (4)
						81 (1)
	19F	1 (2.1)	3 (10.0)	0 (0.0)	4 (3.1)	1421 (3)
						1203 (1)
	6A	2 (4.2)	0 (0.0)	1 (2.0)	3 (2.3)	1876 (1)
						5623 (1)
	3	1 (2.1)	1 (3.3)	0	2 (1.6)	5616 (1)
	23A	0 (0.0)	1 (3.3)	1 (2.0)	3 (1.6)	338 (1)
						439 (1)
	1	0 (0.0)	1 (3.3)	0 (0.0)	1 (0.8)	615 (1)
	4	1 (2.1)	0 (0.0)	0 (0.0)	1 (0.8)	206 (1)
	9V	0 (0.0)	1 (3.3)	0 (0.0)	1 (0.8)	280 (1)
	7F	0 (0.0)	0 (0.0)	0 (0.0)	0 (0.0)	0 (0)
Non-vaccine serotypes		6 (12.5)	3 (10.0)	15 (30.0)	24 (18.8)	Other (7,8%)^d^

a Ranked by frequency of total vaccine serotypes present in PCV13+24F.

b STs were identified in 116 of the 128 samples. The most frequent STs are shown in bold.

c p<0.001 for the comparison of ENI-1, ENI-2, and ENI-3 using the Chi-square test of independence.

d Others: ST4063, ST1292, ST5453, ST5456, ST7441, ST5472, ST5468, ST5475, ST230.

During the pre-PCV7 study, 64.5% (31/48) of pneumonia cases were caused by serotypes covered by PCV7, while in the post-PCV13 period, no cases of pneumonia caused by serotypes covered by PCV7 were reported. Serotypes not included in PCV13 increased from 12.5% (6/48) to 30% (15/50) (supplementary figure 1).

With regard to antibiotic resistance, we found that resistance to penicillin and ceftriaxone was low (6.0% and 2.0% in IPD 3), while resistance to azithromycin increased from 27.0% to 92.0%, resistance to chloramphenicol increased from 14.6% to 26.0%, clindamycin resistance increased from 18.8% to 84.0%, and cotrimoxazole resistance also increased from 70.8% to 84.0% between the first and last periods (Table 3 and Supplementary Figure 2).

Complicated pneumonia occurred frequently in children <5 years of age (96%), mainly associated with serotype 19A (60%) (Table 4). We also found that 75% of the cases of empyema were caused by serotype 19A; penicillin resistance was 21.4% (6/28) vs. 7% (7/100) in cases without empyema (p<0.05). Similarly, resistance to ceftriaxone was 14.3% (4/28) in empyema cases vs. 4% (4/100) in cases without empyema (p<0.05). No association was found between case fatality and serotypes (Table 4).

**Table 3:** Antibiotic resistance of *Streptococcus pneumoniae* isolated from pediatric patients diagnosed with pneumonia ^a^.

Antibiotic	Resistance			p-value
	IPD-1 n=48 n (%)	IPD-2 n=30 n (%)	IPD-3 n=50 n (%)	
Azithromycin ^b^	13 (27.1)	12 (40.0)	46 (92.0)	<0.001 ^c^
Clindamycin	9 (18.8)	5 (16.7)	42 (84.0)	<0.001 ^c^
Tetracycline	14 (29.2)	9 (30.0)	42 (84.0)	<0.001 ^c^
TMP-SMX	34 (70.8)	20 (66.7)	42 (84.0)	<0.001 ^c^
Chloramphenicol	7 (14.6)	3 (10.0)	13 (26.0)	0.146 ^c^
Penicillin ^d^	2 (4.2)	0 (0.0)	3 (6.0)	
Ceftriaxone ^d^	0 (0.0)	0 (0.0)	1 (2.0)	
Vancomycin	0 (0.0)	0 (0.0)	0 (0.0)	
Rifampicin	0 (0.0)	0 (0.0)	0 (0.0)	
Levofloxacin	ND	0 (0.0)	0 (0.0)	
Linezolid	ND	ND	0 (0.0)	

Susceptibility to penicillin, ceftriaxone, azithromycin, and chloramphenicol determined by the minimum inhibitory concentration (MIC) method and to clindamycin only during IPD-3.

Susceptibility to clindamycin, TMP/SMX, tetracycline, levofloxacin, vancomycin, rifampicin, and linezolid determined by the Kirby-Bauer method.

ND: data not collected, TMP/SMX: trimethoprim/sulfamethoxazole.

a Ranked based on frequency in the IPD-3 period.

b During IPD-1, susceptibility to macrolides was tested with erythromycin.

c p-value derived from the chi-square test of independence.

d Includes non-meningitis cut-off points for penicillin (MIC ≥ 8) and ceftriaxone (MIC ≥ 4).

**Table 4:** Complicated pneumonia and case fatality by age, serotype, and resistance.

		Complicated n=50	Non-complicated n=78	Empyema n=28	Non-empyema n=100	Lethality n=10	Non-lethality n=118
Age group (n=128)							
	≤ 2 years	20 (40.0)	47 (60.3)	10 (35.7)	57 (57.0)	5 (50.0)	62 (52.5)
	3-5 years	28 (56.0)	20 (25.6)	17 (60.7)	31 (31.0)	5 (50.0)	43 (36.4)
	≥6 years	2 (4.0)	11 (14.1)	1 (3.6)	12 (12.0)	0 (0.0)	13 (11.1)
Serotypes ^a^							
	19A	30 (60.0) ^b^	12 (15.4) ^b^	21 (75.0) ^b^	21 (21.0) ^b^	3 (30.0)	39 (33.1)
	14	3 (6.0)	20 (25.6)	1 (3.6)	22 (22.0)	2 (20.0)	21 (17.8)
	6B	5 (10.0)	11 (14.1)	1 (3.6)	15 (15.0)	1 (10.0)	15 (12.7)
	24F	6 (12.0)	5 (6.4)	4 (14.3)	7 (7.0)	0 (0.0)	11 (9.3)
Resistance ^c^ (n=21)							
	Penicillin	8 (16.0)	5 (6.4)	6 (21.4)	7 (7.0)	2 (20.0)	11 (9.3)
	Ceftriaxone	5 (10.0)	3 (3.8)	4 (14.3)	4 (4.0)	1 (10.0)	7 (5.9)

a Most common serotypes

b p<0.001 using the Chi-square test of independence.

c Antibiotic resistance considering intermediate and resistant strains for each non-meningitis cutoff point (penicillin minimum inhibitory concentration, MIC ≥ 4, and for ceftriaxone, MIC ≥ 2).

## DISCUSSION

This study evaluated cases of pneumococcal pneumonia in pediatric patients hospitalized during three periods, analyzing changes in epidemiology, serotypes, and antibiotic resistance. The distribution of pneumonia in the pre- and post-PCV7 study was higher in infants (77.1% and 43.3%) and in the post-PCV13 study in preschoolers (62%) (p<0.001). An increase in the frequency of complicated pneumonia was reported (from 23.3% to 72%). Regarding the distribution of serotypes, we found that serotypes 14 and 6B decreased. In contrast, we also found an increase in serotype 19A (from 8.3% to 68%) and the emergence of serotype 24F (from 0% to 22%). Resistance to penicillin and ceftriaxone remained low (6% and 2%, respectively), while resistance to azithromycin increased from 27.1% to 92%.

The highest proportion of pneumonia in the pre- and post-PCV7 study was found in infants (77.1% and 43.3%, respectively), while in the post-PCV13 study it was reported in preschoolers (62%) (p<0.001). This is probably because during the post-PCV13 period younger children were already being routinely vaccinated as part of a mature vaccination schedule; however, preschoolers were not being vaccinated as infants, or did not have a different schedule to catch up with PCV13. In addition, vaccination coverage was inadequate in previous years ^5^^,^^6^.

The study reported a 48.7% increase in the frequency of complicated pneumonia, affecting 96% of children under 5 years of age. This group is generally susceptible and has poorer prognosis ^9^. Although the serotypes associated with complicated pneumonia (1, 3, 7F, 14, and 19A) are covered by PCV13 ^10^, we found that complications increased, possibly due to low vaccination coverage (45.9%) and vaccine failure, as 6 of 9 patients with a complete vaccination schedule had complicated pneumonia caused by serotype 19A. Other studies have reported similar cases in the United States and Australia ^11^^,^^12^. A particular case is serotype 19A, which, despite being covered by PCV13, increased after its introduction, mainly related to sequence type ST320. It has been suggested that the capsular change of serotype 19A could evade the protection of PCV13 ^4^. Therefore, although penicillin remains the treatment of choice for uncomplicated pneumonia, due to higher rates of resistance found in empyema (21.4%), empirical use of ceftriaxone is recommended in these cases.

We found that serotypes 14 and 6B decreased, but serotype 19A (8.3% to 68%) increased significantly. Similar changes have been reported in Colombia, Chile, and Argentina ^13^^-^^15^. Differences in the frequency of vaccine serotypes could be explained by different vaccination schedules. In Peru, the 2+1 schedule is used, while the United States uses the 3+1 schedule. It is hypothesized that the 2+1 schedule may offer shorter protection ^11^.

A systematic review of serotype distribution among pediatric patients diagnosed with IPD in countries that have introduced PCV10 or PCV13 revealed that serotype 24F was one of the most prevalent in Latin America, Europe, and the Western Pacific but not in North America. Not only is it prevalent, but it also has a high capacity to cause invasive disease. In addition, it frequently colonizes the nasopharynx and is associated with a high prevalence of antibiotic resistance ^16^. Because of this, continued surveillance of *S. pneumoniae* serotypes is recommended so that relevant information from different parts of the world can be provided for the design of the next generation of PCV, which will likely contain this serotype, thereby reducing the remaining burden of pneumococcal disease.

Pneumococcal antibiotic resistance is a public health problem, with significant geographical variations. In 2019, reports showed that, worldwide, 30% of pneumococci were resistant to one or more antibiotics ^17^. Resistance to penicillin and ceftriaxone was found to remain low, similar to that reported in a review of 103 studies in Latin America, where penicillin resistance decreased from 28.4% to 6%, and ceftriaxone/cefotaxime resistance decreased from 11.3% to 6.5% ^18^. In contrast, we reported a significant increase in resistance to azithromycin (27.1% to 92%). Increased resistance to macrolides was also found in healthy carriers in Peru (from 33.5% to 50%) ^4^, Brazil (from 12% to 35.3%) ^19^, Colombia (from 3.8% to 34.1%) post-PCV10 ^13^, and Argentina (from 6.6% to 22.7%) post-PCV13 ^15^.

The strength of this study was the collection of data over 14 years using similar methodologies, which allows for an assessment of the potential impact of vaccines in Peru. However, the study has limitations: not all health centers in Lima were included, although the participants cover 80% of the hospitalized pediatric population; complete data were lacking for some variables due to differences in the data collection forms, and some variables were only collected in post-PCV7 studies; only pneumococcal pneumonia with positive isolation was analyzed, which does not represent all cases of pneumonia; and because it is based on passive surveillance, it does not reflect the total burden of the disease. The study does not attempt to assess the real impact of PCV in Peru, as at least 10 years are required to demonstrate the real impact of PCV in a population ^7^. Even so, the study provides useful data on complications, serotypes, and antibiotic resistance in Peruvian children with pneumonia, which are relevant for their management and prevention.

In conclusion, due to the increase in complicated pneumonia cases, increased resistance to certain antibiotics, and the emergence of some vaccine serotypes and the emergence of others, surveillance of *S. pneumoniae* should be implemented throughout the country in order to evaluate the real impact of current and future vaccines introduced in Peru. However, penicillin remains the antibiotic of choice for the treatment of uncomplicated pneumococcal pneumonia in Lima.
