# Supplementary material for: Clinical and microbiological characteristics of pediatric patients hospitalized for pneumococcal pneumonia before and after the introduction of pneumococcal conjugate vaccines
Source: Rev Peru Med Exp Salud Publica. 2025 Mar 12;42(1):63–9. doi: 10.17843/rpmesp.2025.421.13847 (PMC12176018; doi:10.17843/rpmesp.2025.421.13847)
Supplement: Supplementary material. — Available in the electronic version of the RPMESP. [file rpmesp-42-01-13847-s001.docx]

| **Tabla suplementaria 1.** Susceptibilidad antibiótica por método de concentración mínima inhibitoria (MIC) y por Kirby-Bauer/disco para *S. pneumoniae* según el CLSI 2019 (12). | | | | | | |
| --- | --- | --- | --- | --- | --- | --- |
|  | Categorías y puntos de corte de MIC (mg/L) | | | Categorías y puntos de corte según disco (mm) | | |
|  | S | I | R | S | I | R |
| Penicilina (no-MEC)^a^ | ≤ 2 | 4 | ≥ 8 | - | - | - |
| Ceftriaxona (no-MEC)^a^ | ≤ 1 | 2 | ≥ 4 | - | - | - |
| Azitromicina | ≤ 0.5 | 1 | ≥ 2 | ≥18 | 14-17 | ≤ 13 |
| Eritromicina | ≤ 0.25 | 0.5 | ≥ 1 | ≥ 21 | 16-20 | ≤ 15 |
| Clindamicina | ≤ 0.25 | 0.5 | ≥ 1 | ≥19 | 16-18 | ≤ 15 |
| Tetraciclina | ≤ 1 | 2 | ≥ 4 | ≥28 | 25-27 | ≤ 24 |
| Oxacilina | - | - | - | ≥20 | - | - |
| Cloranfenicol | ≤ 4 | - | ≥ 8 | ≥21 | - | ≤ 20 |
| Vancomicina | ≤ 1 | - | - | ≥ 17 | - | - |
| Rifampicina | ≤ 1 | 2 | ≥ 4 | ≥19 | 17-18 | ≤ 16 |
| TMP-SMX | ≤ 0.5/9.5 | 1/19-2/38 | ≥ 4/76 | ≥19 | 16-18 | ≤ 15 |
| Levofloxacino | ≤ 2 | 4 | ≥ 8 | ≥17 | 14-16 | ≤ 13 |
| Linezolid | ≤ 2 | - | - | ≥21 | - | - |
| ^a^ No-MEC, no meningitis | | | | | | |

| **Tabla suplementaria 2. Resistencia antibiótica de los serotipos 19A y 24F post introducción de la PCV13^a^** | | |
| --- | --- | --- |
|  | Resistencia | |
|  | Serotipo 19A  N = 34  n (%) | Serotipo 24F  N = 11  n (%) |
| Azitromicina | 32 (94,1) | 11 (100,0) |
| TMP-SMX | 32 (94,1) | 8 (72,7) |
| Tetraciclina | 30 (88,2) | 10 (91,0) |
| Clindamicina | 29 (85,3) | 11 (100,0) |
| Cloranfenicol | 10 (29,4) | 3 (27,0) |
| Penicilina | 3 (8,8) | 0 |
| Ceftriaxona | 1 (2,9) | 0 |
| ^a^ Ordenado según frecuencia de resistencia del serotipo 19A | | |


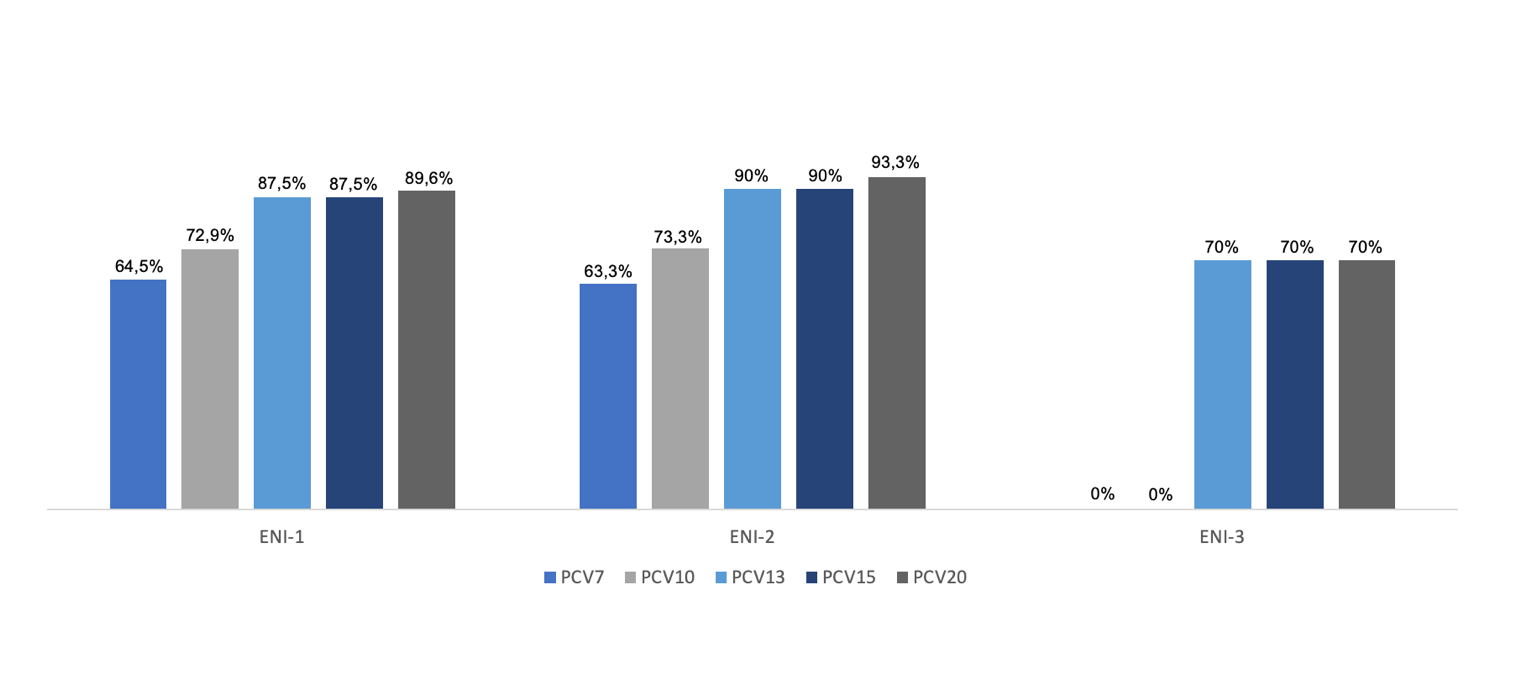
**obse**

**Figura suplementaria 1.** Prevalencia de serotipos incluidos en las vacunas de pacientes pediátricos con neumonía neumocócica durante los estudios ENI-1 (2006-2008), ENI-2 (2009-2011) y ENI-3 (2016-2019). Azul claro PCV7, gris claro PCV10, celeste PCV13, azul oscuro PCV15, gris oscuro PCV20. Serotipos incluidos en la PCV7: 4, 6B, 9V, 14, 18C, 19F, 23F. Serotipos incluido en la PCV10: PCV7 + 1, 5, 7F. Serotipos incluidos en la PCV13: PCV10 + 3, 6A, 19A. Serotipos incluídos em la PCV15: PCV13 + 22F, 33F. Serotipos incluidos en la PCV20: PCV15 + 8, 10A, 11A, 12F, 15B.


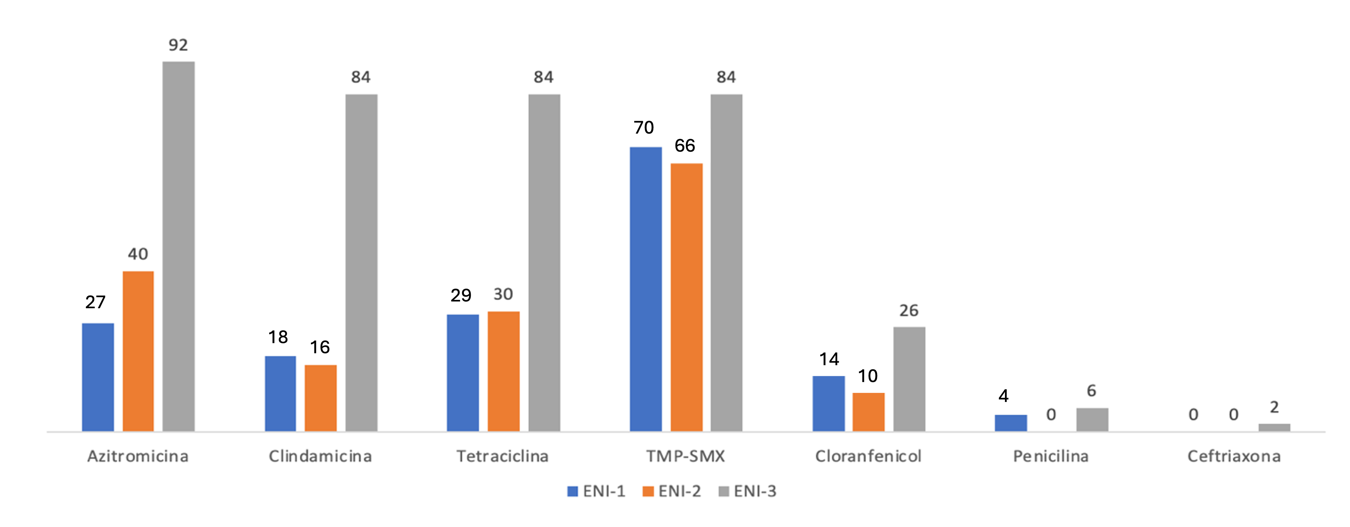


**Figura suplementaria 2.** Porcentaje de resistencia antibiótica de *S.pneumoniae* aislados de pacientes pediátricos con diagnóstico de neumonía durante los estudios ENI-1, ENI-2 y ENI-3. Azul ENI-1 (2006-2008) (n= 48), anaranjado ENI-2 (2009-2011) (n= 30) y gris claro ENI-3 (2016-2019) (n= 50). Susceptibilidad a penicilina, ceftriaxona, azitromicina y cloranfenicol determinada por el método de concentración mínima inhibitoria (MIC) y a clindamicina solo durante el ENI-3. Susceptibilidad a clindamicina, TMP/SMX, tetraciclina, levofloxacina, vancomicina, rifampicina y linezolid determinada por el método Kirby-Bauer. Comprende puntos de corte de no meningitis para penicilina (MIC ≥ 8) y para ceftriaxona (MIC ≥ 4). La resistencia a vancomicina, rifampicina, levofloxacino y linezolid fue cero.

**Tabla suplementaria 3. Definición operacional de variables^a^**

| VARIABLES | DEFINICIÓN | INDICADOR | TIPO DE VARIABLE |
| --- | --- | --- | --- |
| Sexo | Condición orgánica que diferencia a los hombres de las mujeres | Femenino, masculino | Cualitativa nominal dicotómica |
| Edad | Tiempo transcurrido desde el nacimiento del individuo | Dato expresado en meses, años | Cuantitativa discreta |
| Comorbilidades | Presencia de comorbilidades en el paciente (malnutrición, asma, anemia, etc.) | Sí, no | Cualitativa dicotómica |
| Estado de vacunación | Condición de vacunación del individuo | Completa, incompleta, ninguna. | Cualitativa nominal |
| Tipo de vacuna | El tipo de PCV que recibió el paciente | PCV7, PCV10, PCV13, no dato | Cualitativa nominal |
| Lugar de cultivo | Sitio de donde se obtuvo el cultivo positivo a *S.pneumoniae* | Hemocultivo, líquido pleural, ambos | Cualitativa nominal |
| Leucocitos | Valor de leucocitos obtenidos en el hemograma al ingreso | Dato expresado en números | Cuantitativa discreta |
| Segmentados | Valor numérico de segmentados obtenidos en el hemograma de ingreso | Dato expresado en números | Cuantitativa discreta |
| Patrón radiológico | Tipo de infiltrado en la radiografía de tórax | Alveolar, intersticial, alveolar-intersticial | Cualitativa nominal |
| Efusión pleural | Cantidad anormal de líquido en el espacio pleural | Sí, no | Cualitativa dicotómica |
| Empiema | Colección de pus en la cavidad pleural, positivo a gram o cultivo del líquido pleural. | Sí, no | Cualitativa dicotómica |
| Neumonía necrotizante | Complicación severa de neumonía bacteriana donde se evidencia necrosis en el pulmón | Sí, no | Cualitativa dicotómica |
| Sepsis | Individuo presenta signos y síntomas de Síndrome de respuesta inflamatoria sistémica (SIRS) | Sí, no | Cualitativa dicotómica |
| Insuficiencia respiratoria | Individuo necesitó el uso de ventilación mecánica como soporte respiratorio | Sí, no | Cualitativa dicotómica |
| Letalidad | Muerte del individuo | Sí, no | Cualitativa dicotómica |
| Resistencia antibiótica  (Tabla suplementaria 1) | Muestra de *S.pneumoniae* que presenta resistencia antibiótica   - Penicilina por MIC - Ceftriaxona por MIC - Eritromicina o azitromicina por MIC - Clindamicina por MIC - Tetraciclina por MIC - Oxacilina por disco - Cloranfenicol por MIC - Vancomicina por disco - Rifampicina por disco - TMP-SMX por MIC - Levofloxacino por disco - Linezolid por disco - Cloranfenicol por MIC | R, I, S  por cada antibiótico | Cualitativa dicotómica |
| Serotipos | Serotipos identificados en los individuos | 6A, 19A, etc. | Cualitativa nominal |

^a^La forma de registro se obtuvo a partir de datos tomados de los estudios ENI1, ENI2 y ENI3.
