# Supplementary material for: Clinical and microbiological characteristics of pediatric patients hospitalized for pneumococcal pneumonia before and after the introduction of pneumococcal conjugate vaccines
Source: Rev Peru Med Exp Salud Publica. 2025 Mar 12;42(1):63–9. doi: 10.17843/rpmesp.2025.421.13847 (PMC12176018; doi:10.17843/rpmesp.2025.421.13847)
Supplement: Translation

# Translation: Características clínicas y microbiológicas de pacientes pediátricos hospitalizados por neumonía neumocócica antes y después de la introducción de las vacunas conjugadas neumocócicas

**Authors:** Carolina A. Gomez, Brayan E. Gonzales, Roger A. Hernández, Francisco Campos, Eduardo Chaparro, Olguita Del Águila, María E. Castillo, Andrés Saenz, Isabel Reyes, Theresa J. Ochoa

**Affiliations:** 1 Facultad de Medicina Humana «Alberto Hurtado», Universidad Peruana Cayetano Heredia, Lima, Perú.; 2 Instituto de Medicina Tropical «Alexander von Humboldt», Universidad Peruana Cayetano Heredia, Lima, Perú.; 3 Departamento de Pediatría, Hospital Nacional Cayetano Heredia, Lima, Perú.; 4 Hospital Nacional Docente Madre-Niño San Bartolomé. Lima, Perú.; 5 Servicio de Pediatría de Especialidades Clínicas, Hospital Nacional Edgardo Rebagliati Martins, Lima, Perú.; 6 Oficina de Epidemiología, Instituto Nacional de Salud del Niño, Lima, Perú.; 7 Departamento de Pediatría, Hospital Nacional Daniel Alcides Carrión, Lima, Perú.; 8 Servicio de Hospitalización, Hospital de Emergencias Pediátricas, Lima, Perú.

**Keywords:** Neumonía neumocócica, Vacunas neumocócicas, empiema, niños, Lima

## Abstract

Se analizaron las características clínicas y microbiológicas de neumonía neumocócica en niños hospitalizados en Lima, antes y después de la introducción de las vacunas neumocócicas conjugadas (PCV). Se revisaron casos entre 2006-2019, en los periodos pre-PCV7 (2006-2008), post-PCV7 (2009-2011) y post-PCV13 (2016-2019). De 128 pacientes con cultivos positivos (92 de sangre y 36 de líquido pleural), la mayoría fueron lactantes en los estudios pre-PCV7 (77,1%) y post-PCV7 (43,3%), mientras que en el estudio post-PCV13 fueron mayoritariamente preescolares (62%). Se observó un aumento de neumonías complicadas de 14,6% a 72% y un incremento del serotipo 19A (6,3% a 68%). El secuenciotipo más frecuente fue ST320 (31 aislamientos). La resistencia a penicilina y ceftriaxona fue baja (6% y 2%, respectivamente), aunque mayor en empiema (21,4% y 14,3%, respectivamente). La resistencia a azitromicina se incrementó de 27% a 92%. La penicilina sigue siendo el antibiótico de elección para el tratamiento de neumonía neumocócica no complicada en Lima.

## INTRODUCCIÓN

La neumonía es la principal causa de muerte de origen infeccioso en niños menores de cinco años, siendo responsable del 14% de las muertes en este grupo etario. *Streptococcus pneumoniae* o neumococo es la principal causa de neumonía bacteriana en niños ^1^. En el 2015 se reportó 9,18 millones de casos de infección neumocócica a nivel global en población general, con 318 000 casos fatales. El 97% de las infecciones neumocócicas son neumonía, mientras que el 81% de las muertes causadas por neumococo son por neumonía ^2^.

El uso de las vacunas neumocócicas conjugadas (PCV, por sus siglas en inglés), como medida de salud pública, ha cambiado la dinámica de esta enfermedad. En el mundo, del 2000 a 2015 hubo una disminución de 37% de casos de neumonía en niños luego de la introducción de las vacunas ^2^. En el Perú, la primera vacuna en ser usada fue la PCV7 en 2009, la PCV10 en 2011 y finalmente la PCV13 en el 2015 ^3^. La PCV15 y PCV20 aún no están disponibles en Perú. La distribución de serotipos de neumococo varía según la región y las PCV introducidas ^4^.

Estudios realizados en Lima en los últimos 14 años han permitido comparar la resistencia y la distribución de serotipos de enfermedad neumocócica invasiva (ENI). Sin embargo, no se realizó un análisis enfocado en los casos de neumonía específicamente. El objetivo del presente estudio es describir y comparar las características clínicas, demográficas y microbiológicas de pacientes pediátricos con neumonía neumocócica antes y después de la introducción de las PCV en Lima, Perú.

**MENSAJE CLAVE**

Motivación para realizar el estudio. Si bien, en Lima se han llevado a cabo estudios sobre la enfermedad neumocócica invasiva, se desconocen las características de neumonía por neumococo antes y después de la introducción de las vacunas neumocócicas conjugadas.

Principales hallazgos. Se encontró un aumento de neumonía neumocócica complicada, especialmente empiema y aumento del serotipo 19A. La resistencia a penicilina y ceftriaxona se mantiene baja; sin embargo, en casos de empiema, la resistencia antibiótica fue mayor.

Implicancias en salud pública. Se debe continuar indicando penicilina o amoxicilina para el manejo empírico de neumonía neumocócica; sin embargo, para casos con empiema se sugiere usar ceftriaxona. Se recomienda seguir monitorizando la resistencia y los serotipos de neumococo localmente.

## EL ESTUDIO

### Diseño del estudio

Estudio descriptivo de análisis secundario de bases de datos, obtenidos del registro clínico de tres estudios multicéntricos de vigilancia pasiva de ENI realizado por el Grupo Peruano de Investigación de Neumococo (GPIN) en los periodos 2006-2008 [pre-PCV7(ENI-1)] ^5^, 2009-2011 [post-PCV7(ENI-2)] ^6^ y 2016-2019 [post-PCV13(ENI-3)] ^7^. Los estudios se realizaron en 11 hospitales públicos y 7 clínicas y laboratorios privados de Lima Metropolitana.

Estos estudios recolectaron información clínica, resistencia antibiótica y genómica. Se empleó el método E-test para determinar la concentración mínima inhibitoria (MIC en inglés) y Kirby-Bauer para 12 antibióticos, usando los puntos de corte establecidos por el CLSI-2019 ^8^ (tabla suplementaria 1). Los serotipos y secuenciotipos (ST) se determinaron por secuenciameinto del genoma completo (WGS, *whole genome sequencing*).

### Población

La población estuvo conformada por pacientes menores de 18 años hospitalizados en Lima con el diagnóstico de neumonía neumocócica.

### Definición de caso

Se definió neumonía neumocócica como un aislamiento de *S. pneumoniae* en hemocultivo o cultivo de líquido pleural (LP) en pacientes con un proceso infeccioso con fiebre, dificultad respiratoria y/o evidencia de infiltrados pulmonares en la radiografía de tórax. Se definió neumonía complicada en el contexto de neumonía neumocócica donde se agregan complicaciones locales (efusión pleural, empiema, neumonía necrotizante, insuficiencia respiratoria con requerimiento de ventilación mecánica) o complicación sistémica (sepsis o muerte) ^6^.

### Variables

Las variables se describen en la tabla suplementaria 3.

### Análisis estadístico

Las variables categóricas se muestran con frecuencias absolutas y relativas, y las variables continuas con medianas y rango intercuartílicos (RIC). Para determinar las diferencias entre los estudios de ENI se utilizó la prueba de Chi cuadrado de independencia o de Fisher, según corresponda. En las variables continuas, se empleó la prueba *Kruskall-Wallis.* Todos los análisis se realizaron en STATA SE versión 18.

### Consideraciones éticas

Los estudios de ENI tuvieron la aprobación del Comité de Ética en Investigación de la Universidad Peruana Cayetano Heredia (UPCH) y de cada hospital participante. El presente estudio tuvo la aprobación del Comité de Ética en Investigación de la UPCH (SIDISI 211822).

## RESULTADOS

Se identificaron 128 casos de neumonía por neumococo en pacientes pediátricos, 48 en el período pre-PCV7, 30 en post-PCV7 y 50 en post-PCV13. En los dos primeros períodos hubo más casos de neumonía neumocócica en lactantes (77,1% y 43,3% respectivamente), en el último más en preescolares (62%). El 71,9% del total de cepas fueron aisladas de hemocultivos y 28,1% de LP; el aislamiento en LP aumentó de 14,6% a 48%. En el período post-PCV7 solo 16,7% estuvieron vacunados, mientras que en el estudio post-PCV13 el 70% había recibido la PCV10 o PCV13 (tabla 1).

**Tabla 1:** Características demográficas, clínicas y de laboratorio de pacientes pediátricos con neumonía neumocócica ^a^.

Características	ENI-1 n (%)	ENI-2 n (%)	ENI-3 n (%)	Total n (%)
Sexo, masculino (n=128)	28 (62,2)	16 (53,3)	22 (44,0)	66 (52,8)
Edad, años ^b^, mediana (RIC)	1 (1-2)	2 (1-7)	2 (1-3)	1,5 (1-3)
Grupo etario (n=128)				
Lactantes (<2 años)	37 (77,1)	13 (43,3)	17 (34,0)	67 (52,3)
Preescolares (2-6 años)	10 (20,8)	7 (23,3)	31 (62,0)	48 (37,5)
Escolares (>6 años)	1 (2,1)	10 (33,3)	2 (4,0)	13 (10,2)
Comorbilidad (n=40)	ND	16 (53,3)	24 (48,0)	40 (50,0)
Malnutrición	ND	8 (26,7)	8 (16,0)	16 (20,0)
Asma	ND	4 (13,3)	3 (6,0)	7 (8,8)
Anemia	ND	1 (6,3)	5 (10,0)	6 (7,5)
Otros ^c^	ND	3 (3,3)	6 (12,0)	11 (13,8)
Estado de vacunación con PCV (n=120)				
Completa	0	2 (6,7)	25 (50,0)	27 (21,1)
Incompleta	0	3 (10,0)	14 (28,0)	17 (13,3)
Ninguna	48 (100,0)	22 (73,3)	6 (12,0)	76 (59,4)
Tipo de vacuna (n=118)				
PCV13	0	0	21 (42,0)	21 (16,4)
PCV10	0	0	14 (28,0)	14 (10,9)
PCV7	0	5 (16,7)	2 (4,0)	7 (5,5)
Ninguna	48 (100,0)	22 (73,3)	6 (12,0)	76 (59,4)
Lugar de cultivo (n=128)				
Sangre	41 (85,4)	25 (83,3)	26 (52,0)	92 (71,9)
Líquido pleural	7 (14,6)	5 (16,7)	24 (48,0)	36 (28,1)
Patrón radiológico (n=67)				
Alveolar	ND	15 (57,7)	34 (82,9)	49 (73,1)
Alveolo-intersticial	ND	10 (38,5)	4 (9,8)	14 (20,9)
Intersticial	ND	1 (3,9)	3 (7,3)	4 (6,0)
Pruebas de laboratorio				
Leucocitos, mediana (RIC)	ND	7200 (3060-17 200)	6000 (3020-14 500)	6000 (3060-15 000)
Segmentados %, mediana (RIC)	ND	76 (64,75-87,25)	80 (75-85)	80 (70-90)
Complicaciones ^d,e^ (n=50)	7 (14,6)	7 (23,3)	36 (72,0)	50 (39,1)
Empiema	ND	3 (10,0)	25 (50,0)	28 (35,0)
Sepsis	ND	2 (6,7)	17 (34,0)	19 (23,8)
Insuficiencia respiratoria	ND	2 (6,7)	17 (34,0)	19 (23,8)
Neumonía necrotizante	ND	1 (3,3)	13 (26,0)	14 (17,5)
Efusión pleural	ND	1 (3,3)	6 (12,0)	7 (8,8)
Letalidad (n=10)	7 (14,6)	0 (0,0)	3 (6,0)	10 (7,8)^f^

a Algunas variables suman menos de 128 debido a valores perdidos, por esta razon algunos porcentajes no suman 100%.

b p<0,001, para la comparación de ENI-1, ENI-2 y ENI-3 usando prueba de *Kruskall-Wallis.*

c Otras comorbilidades menos frecuentes: síndrome obstructivo bronquial, cáncer, cardiopatía congénita, enfermedad respiratoria crónica, prematuridad, obesidad, VIH. Los porcentajes de la columna total están calculados con base en el total de pacientes de ENI-2 y ENI-3.

d Un paciente puede tener más de una complicación, por lo que los porcentajes no suman exactamente 100%. Las presentaciones más frecuentes fueron seis pacientes con empiema, neumonía necrotizante, insuficiencia respiratoria por requerimiento de ventilación mecánica y cuatro pacientes con empiema e insuficiencia respiratoria por requerimiento de ventilación mecánica. Para la distribución de complicaciones en la columna del total, solo se consideraron las muestras de ENI-2 y ENI-3 que tenían datos del tipo de complicación (denominador n=80).

e p<0,001, para la comparación de ENI-1, ENI-2 y ENI-3 usando prueba de Chi cuadrado de independencia.

ND: No se recolectó el dato durante el estudio, RIC: rango intercuartílico, PCV: vacunas neumocócicas conjugadas

f Porcentaje calculado del total=128

A pesar de que en el período pre-PCV7 no se recolectaron datos de complicaciones, se identificaron 7 pacientes con efusión pleural debido a que el aislamiento de neumococo fue de LP. Con respecto a las complicaciones, se observó un aumento de neumonías complicadas de 14,6% a 72%. La complicación más frecuente fue empiema (35%), seguido de sepsis e insuficiencia respiratoria con requerimiento de ventilación mecánica. Entre los 36 casos de neumonías neumocócicas post-PCV13, 25% fueron en pacientes con vacunación completa y 6 casos fueron por el serotipo 19A (tabla 1).

La frecuencia del serotipo 19A incrementó de 6,3%, 13,3% y 68,0%. El serotipo 24F no estuvo presente en los primeros dos períodos, mientras en post-PCV13 representó el 22% de los aislamientos. Además, se observó una disminución del serotipo 14 (31,3%, 26,7% y 0%). Entre todos los aislamientos, los secuenciotipos más frecuentes fueron el ST320 (26,7%) y ST156 (12,9%) (tabla 2).

**Tabla 2:** Distribución de serotipos y secuenciotipos (ST) presentes en las vacunas conjugadas en pacientes con neumonía neumocócica (N = 128) ^a^.

	ENI-1 n=48 n (%)	ENI-2 n=30 n (%)	ENI-3 n=50 n (%)	Total n=128 n (%)	ST n=116^b^ ST (n)
Serotipos					
19A^c^	4 (8,3)	4 (13,3)	34 (68,0)	42 (32,3)	320 (31) 276 (3) 1131 (2) 1451 (2) 5460 (1) 6048 (1)
14	15 (31,3)	8 (26,7)	0 (0,0)	23 (18,0)	156 (15) 15 (2) 6144 (1) 7432 (1) 9054 (1)
6B	12 (25,0)	4 (13,3)	0 (0,0)	16 (12,5)	90 (4) 5625 (3) 1121 (2) 135 (2) 1662 (1) 5449 (1) 5619 (1)
24F	0 (0,0)	0 (0,0)	11 (22,0)	11 (8,6)	230 (9)
5	4 (8,3)	2 (6,7)	0 (0,0)	6 (4,7)	289 (6)
23F	2 (4,2)	3 (10,0)	0 (0,0)	5 (4,0)	242 (4)
					81 (1)
19F	1 (2,1)	3 (10,0)	0 (0,0)	4 (3,1)	1421 (3)
					1203 (1)
6A	2 (4,2)	0 (0,0)	1 (2,0)	3 (2,3)	1876 (1)
					5623 (1)
3	1 (2,1)	1 (3,3)	0	2 (1,6)	5616 (1)
23A	0 (0,0)	1 (3,3)	1 (2,0)	3 (1,6)	338 (1)
					439 (1)
1	0 (0,0)	1 (3,3)	0 (0,0)	1 (0,8)	615 (1)
4	1 (2,1)	0 (0,0)	0 (0,0)	1 (0,8)	206 (1)
9V	0 (0,0)	1 (3,3)	0 (0,0)	1 (0,8)	280 (1)
7F	0 (0,0)	0 (0,0)	0 (0,0)	0 (0,0)	0 (0)
Serotipos no vacunales	6 (12,5)	3 (10,0)	15 (30,0)	24 (18,8)	Otros (7,8%)^d^

a Ordenados por frecuencia del total de serotipos vacunales presentes en la PCV13+24F.

b Se identificaron los ST en 116 de las 128 muestras. En negrita los ST más frecuentes.

c p<0,001 para la comparación de ENI-1, ENI-2 y ENI-3 usando la prueba de Chi cuadrado de independencia.

d Otros: ST4063, ST1292, ST5453, ST5456, ST7441, ST5472, ST5468, ST5475, ST230.

Durante el estudio pre-PCV7, 64,5% (31/48) de los casos de neumonía fueron por serotipos cubiertos por la PCV7; mientras que en el período post-PCV13 no se reportaron casos de neumonía por serotipos cubiertos por las PCV7. Los serotipos no incluidos en la PCV13 aumentaron de 12,5% (6/48) a 30% (15/50) (figura 1 suplementaria).

Con respecto a la resistencia antibiótica, la resistencia a penicilina y ceftriaxona fue baja (6,0% y 2,0% en ENI 3), mientras que la resistencia a azitromicina aumentó de 27,0% a 92,0%, resistencia a cloranfenicol de 14,6% a 26,0%, resistencia a clindamicina de 18,8% a 84,0% y resistencia a cotrimoxazol de 70,8% a 84,0%, entre el primer y último período, respectivamente (tabla 3 y figura 2 suplementaria).

**Tabla 3:** Resistencia antibiótica de *Streptococcus pneumoniae* aislados de pacientes pediátricos con diagnóstico de neumonía ^a^.

Antibiótico	Resistencia			Valor de p
	ENI-1 n=48 n (%)	ENI-2 n=30 n (%)	ENI-3 n=50 n (%)	
Azitromicina ^b^	13 (27,1)	12 (40,0)	46 (92,0)	<0,001 ^c^
Clindamicina	9 (18,8)	5 (16,7)	42 (84,0)	<0,001 ^c^
Tetraciclina	14 (29,2)	9 (30,0)	42 (84,0)	<0,001 ^c^
TMP-SMX	34 (70,8)	20 (66,7)	42 (84,0)	<0,001 ^c^
Cloranfenicol	7 (14,6)	3 (10,0)	13 (26,0)	0,146 ^c^
Penicilina ^d^	2 (4,2)	0 (0,0)	3 (6,0)	
Ceftriaxona ^d^	0 (0,0)	0 (0,0)	1 (2,0)	
Vancomicina	0 (0,0)	0 (0,0)	0 (0,0)	
Rifampicina	0 (0,0)	0 (0,0)	0 (0,0)	
Levofloxacino	ND	0 (0,0)	0 (0,0)	
Linezolid	ND	ND	0 (0,0)	

Susceptibilidad a penicilina, ceftriaxona, azitromicina y cloranfenicol determinada por el método de concentración mínima inhibitoria (MIC) y a clindamicina solo durante el ENI-3.

Susceptibilidad a clindamicina, TMP/SMX, tetraciclina, levofloxacina, vancomicina, rifampicina y linezolid determinada por el método Kirby-Bauer.

ND: no se recolectó el dato, TMP/SMX: trimetoprima/sulfametoxazol.

a Ordenado en base a frecuencia en el periodo ENI-3.

b Durante ENI-1 se hizo la susceptibilidad a macrólidos con eritromicina.

c Valor de p originado por prueba de Chi cuadrado de independencia.

d Comprende puntos de corte de no meningitis para penicilina (MIC ≥ 8) y para ceftriaxona (MIC ≥ 4).

Las neumonías complicadas se presentaron frecuentemente en niños <5 años (96%), asociado principalmente al serotipo 19A (60%) (tabla 4). En los casos de empiema el 75% fue por serotipo 19A; la resistencia a penicilina fue 21,4% (6/28) vs. 7% (7/100) en los casos sin empiema (p<0,05). Igualmente, la resistencia a ceftriaxona fue 14,3% (4/28) en empiema vs. 4% (4/100) en casos sin empiema (p<0,05). No se encontró asociación entre la letalidad y los serotipos (tabla 4).

**Tabla 4:** Neumonía complicada y letalidad según edad, serotipo y resistencia.

	Complicada n=50	No complicada n=78	Empiema n=28	No empiema n=100	Letalidad n=10	No letalidad n=118
Grupo etario (n=128)						
≤ 2 años	20 (40,0)	47 (60,3)	10 (35,7)	57 (57,0)	5 (50,0)	62 (52,5)
3-5 años	28 (56,0)	20 (25,6)	17 (60,7)	31 (31,0)	5 (50,0)	43 (36,4)
≥6 años	2 (4,0)	11 (14,1)	1 (3,6)	12 (12,0)	0 (0,0)	13 (11,1)
Serotipos ^a^						
19A	30 (60,0) ^b^	12 (15,4) ^b^	21 (75,0) ^b^	21 (21,0) ^b^	3 (30,0)	39 (33,1)
14	3 (6,0)	20 (25,6)	1 (3,6)	22 (22,0)	2 (20,0)	21 (17,8)
6B	5 (10,0)	11 (14,1)	1 (3,6)	15 (15,0)	1 (10,0)	15 (12,7)
24F	6 (12,0)	5 (6,4)	4 (14,3)	7 (7,0)	0 (0,0)	11 (9,3)
Resistencia ^c^ (n=21)						
Penicilina	8 (16,0)	5 (6,4)	6 (21,4)	7 (7,0)	2 (20,0)	11 (9,3)
Ceftriaxona	5 (10,0)	3 (3,8)	4 (14,3)	4 (4,0)	1 (10,0)	7 (5,9)

a Serotipos más frecuentes

b p<0,001 utilizando la prueba de Chi cuadrado de independencia.

c Resistencia antibiótica que considera los intermedios y resistentes para cada punto de corte de no meningitis (penicilina concentración mínima inhibitoria, MIC ≥ 4 y para ceftriaxona MIC ≥ 2).

## DISCUSIÓN

Este estudio evaluó los casos de neumonía neumocócica de pacientes pediátricos hospitalizados en tres períodos, analizando los cambios en la epidemiología, serotipos y resistencia antibiótica. La distribución de las neumonías en el estudio pre y post-PCV7 fue mayor en lactantes (77,1% y 43,3%) y en el estudio post-PCV13 en preescolares (62%) (p<0,001). Se observó un aumento en la frecuencia de neumonías complicadas (de 23,3% a 72%). En cuanto a la distribución de serotipos, se identificó una disminución en los serotipos 14 y 6B. En contraste, se observó un aumento del serotipo 19A (de 8,3% a 68%) y la emergencia del serotipo 24F (de 0% a 22%). En relación con la resistencia antibiótica, la resistencia a penicilina y ceftriaxona se mantuvo baja (6% y 2% respectivamente) mientras que la resistencia a azitromicina aumentó de 27,1% a 92%.

La mayor proporción de neumonías en el estudio pre y post-PCV7 se observó en lactantes (77,1% y 43,3%, respectivamente), mientras que en el estudio post-PCV13 fue en preescolares (62%) (p<0,001). Probablemente esto se deba a que en el período post-PCV13 los niños más pequeños ya están siendo rutinariamente vacunados como parte de un esquema de vacunación maduro; sin embargo, los preescolares no han sido vacunados de lactantes, o no han recibido un esquema distinto para ponerse al día con la PCV13. Adicionalmente, la cobertura vacunal no fue adecuada en los años previos ^5^^,^^6^.

El estudio reportó un aumento de 48,7% en la frecuencia de neumonías complicadas, afectando al 96% de los niños <5 años. Este grupo es generalmente susceptible y tienen peor pronóstico ^9^. Aunque los serotipos asociados a neumonía complicada (1, 3, 7F, 14 y 19A) están cubiertos por la PCV13 ^10^, se observó un aumento de complicaciones, posiblemente debido a una baja cobertura vacunal (45,9%) y fallas en la vacuna, ya que 6 de 9 pacientes con esquema completo presentaron neumonía complicada por el serotipo 19A. Otros estudios han reportado casos similares en Estados Unidos y Australia ^11^^,^^12^. Un caso particular es el serotipo 19A que, pese a que estaba cubierto por la PCV13, se incrementó luego de su introducción, principalmente relacionada con el secuenciotipo ST320. Se sugiere que el cambio capsular del serotipo 19A podría evadir la protección de la PCV13 ^4^. Por lo tanto, aunque la penicilina sigue siendo el tratamiento de elección para neumonía no complicada, debido a mayores porcentajes de resistencia encontrados en empiema (21,4%), se recomienda el uso empírico de ceftriaxona en estos casos.

Se identificó una disminución en los serotipos 14 y 6B, pero un aumento significativo del serotipo 19A (8,3% a 68%). En Colombia, Chile y Argentina se han reportado cambios similares ^13^^-^^15^. Las diferencias en la frecuencia de serotipos vacunales podrían explicarse por los distintos esquemas de vacunación. En Perú se usa el esquema 2+1, mientras que en Estados Unidos se usa el 3+1. Se hipotetiza que el esquema 2+1 podría ofrecer una protección más corta ^11^.

Una revisión sistemática de la distribución de serotipos entre pacientes pediátricos con diagnóstico de ENI en países que han introducido la PCV10 o PCV13 reveló que el serotipo 24F fue uno de los más prevalentes en Latinoamérica, Europa y el Pacífico Occidental pero no en Norteamérica. No solo es prevalente, sino que presenta una alta capacidad de causar enfermedad invasiva. Además, coloniza frecuentemente la nasofaringe y está asociado a una elevada prevalencia de resistencia antibiótica ^16^. Debido a esto, se recomienda continuar con la vigilancia de serotipos de *S. pneumoniae,* de tal manera que se pueda proporcionar información relevante de diferentes partes del mundo para el diseño de la próxima generación de PCV, que probablemente contenga a este serotipo, y así disminuir la carga restante de la enfermedad neumocócica.

La resistencia antibiótica de neumococo es un problema de salud pública, con variaciones geográficas importantes. En 2019, se reportó a nivel global que el 30% de los neumococos presentaban resistencia a uno o más antibióticos ^17^. Se encontró que la resistencia a penicilina y ceftriaxona se mantuvo baja, similar a lo reportado en una revisión de 103 estudios en Latinoamérica, donde la resistencia a penicilina disminuyó de 28,4% al 6%, y la resistencia a ceftriaxona/cefotaxime disminuyó de 11,3% al 6,5% ^18^. En contraste, reportamos un aumento importante de la resistencia a azitromicina (27,1% a 92%). El incremento de resistencia a macrólidos también se encontró en portadores sanos en Perú (de 33,5% a 50%) ^4^, Brasil (de 12% a 35,3%) ^19^, Colombia (de 3,8% a 34,1%) post-PCV10 ^13^^)^ y Argentina (de 6,6% a 22,7%) post-PCV13 ^(^^15^.

La fortaleza del estudio fue la recolección de datos durante 14 años con metodologías similares, lo que permite evaluar el impacto potencial de las vacunas en el Perú. Sin embargo, el estudio tiene limitaciones: no se incluyeron todos los centros de salud de Lima, aunque los participantes cubren el 80% de la población pediátrica hospitalizada; faltaron datos completos en algunas variables debido a diferencias en las fichas de recolección, así como algunas variables solo se colectaron en los estudios post-PCV7; solo se analizaron neumonías neumocócicas con aislamiento positivo, lo que no representa todas las neumonías, y al basarse en vigilancia pasiva, no refleja la carga total de la enfermedad. El estudio no intenta evaluar un real impacto de las PCV en el Perú, ya que se requiere un tiempo de al menos 10 años para evidenciar el real impactos de las PCV en una población ^7^. Aun así, el estudio aporta datos útiles sobre complicaciones, serotipos y resistencia antibiótica en niños peruanos con neumonía, relevantes para su manejo y prevención.

En conclusión, debido al incremento de neumonías complicadas, incremento de resistencia a ciertos antibióticos e incremento de algunos serotipos vacunales y emergencia de otros, se recomienda implementar la vigilancia de *S. pneumoniae* en todo el país, a fin de poder evaluar el real impacto de las actuales y futuras vacunas introducidas en el Perú. Sin embargo, la penicilina sigue siendo el antibiótico de elección para el tratamiento de neumonía neumocócica no complicada en Lima.
